# Congenital hypothyroidism

**DOI:** 10.1186/1750-1172-5-17

**Published:** 2010-06-10

**Authors:** Maynika V Rastogi, Stephen H LaFranchi

**Affiliations:** 1 Department of Pediatrics, Division of Endocrinology, Oregon Health & Science University,707 SW Gaines St., Portland, OR. USA

## Abstract

Congenital hypothyroidism (CH) occurs in approximately 1:2,000 to 1:4,000 newborns. The clinical manifestations are often subtle or not present at birth. This likely is due to trans-placental passage of some maternal thyroid hormone, while many infants have some thyroid production of their own. Common symptoms include decreased activity and increased sleep, feeding difficulty, constipation, and prolonged jaundice. On examination, common signs include myxedematous facies, large fontanels, macroglossia, a distended abdomen with umbilical hernia, and hypotonia. CH is classified into permanent and transient forms, which in turn can be divided into primary, secondary, or peripheral etiologies. Thyroid dysgenesis accounts for 85% of permanent, primary CH, while inborn errors of thyroid hormone biosynthesis (dyshormonogeneses) account for 10-15% of cases. Secondary or central CH may occur with isolated TSH deficiency, but more commonly it is associated with congenital hypopitiutarism. Transient CH most commonly occurs in preterm infants born in areas of endemic iodine deficiency. In countries with newborn screening programs in place, infants with CH are diagnosed after detection by screening tests. The diagnosis should be confirmed by finding an elevated serum TSH and low T4 or free T4 level. Other diagnostic tests, such as thyroid radionuclide uptake and scan, thyroid sonography, or serum thyroglobulin determination may help pinpoint the underlying etiology, although treatment may be started without these tests. Levothyroxine is the treatment of choice; the recommended starting dose is 10 to 15 mcg/kg/day. The immediate goals of treatment are to rapidly raise the serum T4 above 130 nmol/L (10 ug/dL) and normalize serum TSH levels. Frequent laboratory monitoring in infancy is essential to ensure optimal neurocognitive outcome. Serum TSH and free T4 should be measured every 1-2 months in the first 6 months of life and every 3-4 months thereafter. In general, the prognosis of infants detected by screening and started on treatment early is excellent, with IQs similar to sibling or classmate controls. Studies show that a lower neurocognitive outcome may occur in those infants started at a later age (> 30 days of age), on lower l-thyroxine doses than currently recommended, and in those infants with more severe hypothyroidism.

## Definition and classification

Congenital hypothyroidism (CH) is defined as thyroid hormone deficiency present at birth. Thyroid hormone deficiency at birth is most commonly caused by a problem with thyroid gland development (dysgenesis) or a disorder of thyroid hormone biosynthesis (dyshormonogenesis). These disorders result in primary hypothyroidism. Secondary or central hypothyroidism at birth results from a deficiency of thyroid stimulating hormone (TSH). Congenital TSH deficiency may rarely be an isolated problem (caused by mutations in the TSH β subunit gene), but most commonly it is associated with other pituitary hormone deficiencies, as part of congenital hypopituitarism. Peripheral hypothyroidism is a separate category resulting from defects of thyroid hormone transport, metabolism, or action.

Congenital hypothyroidism is classified into permanent and transient CH. Permanent CH refers to a persistent deficiency of thyroid hormone that requires life-long treatment. Transient CH refers to a temporary deficiency of thyroid hormone, discovered at birth, but then recovering to normal thyroid hormone production. Recovery to euthyroidism typically occurs in the first few months or years of life. Permanent CH can be further classified into permanent primary and secondary (or central) CH; transient primary CH has also been reported. In addition, some forms of CH are associated with defects in other organ systems; these are classified as syndromic hypothyroidism.

The underlying etiology of CH typically will determine whether hypothyroidism is permanent or transient, primary, secondary, or peripheral, and whether there is involvement of other organ systems (see section on Etiology for details). The primary emphasis of this review is a discussion of primary CH, but there also will be some discussion of secondary or central CH. It should be borne in mind that an underlying etiology may not be determined for many cases of CH. Further, while the exact cause of some cases of thyroid dysgenesis is known, e.g., a mutation in the *TTF-2 *gene, mutations in genes encoding such transcription factors important in thyroid gland development have been found in only 2% of cases. Thus, an exact cause for the vast majority of cases of thyroid dysgenesis remains unknown. This has not been a significant issue, however, as management of CH is based on restoring thyroid function to normal, not necessarily knowing the exact underlying cause.

## Epidemiology

Prior to the onset of newborn screening programs, the incidence of congenital hypothyroidism, as diagnosed after clinical manifestations, was in the range of 1;7,000 to 1:10,000 [1]. With the advent of screening of newborn populations, the incidence was initially reported to be in the range of 1:3,000 to 1:4,000 [2]. With more experience from state, regional, and national screening programs, it has become apparent that the incidence varies by geographic location. A report from the French newborn screening program summarizing a 20 year period found the incidence of permanent hypothyroidism to be 1:10,000 [3], whereas a report from the Greek Cypriot population over an 11 year period found the incidence in newborns to be 1:800 [4].

A recent report showed that the incidence in the United States increased from 1:4,094 in 1987 to 1:2,372 in 2002 [5]. The reason(s) for the increased incidence is not clear, but one possible explanation may be a change in testing strategy. With increased sensitivity and accuracy of TSH methods, many U.S. and other programs around the world have switched from a primary T4-follow-up TSH approach to a primary TSH test. If the TSH cutoff is lowered, more infants with milder congenital hypothyroidism will be detected. In addition, there is some variation in the incidence among different racial and ethnic groups, and the mix of these groups has changed. Several U.S. programs have reported a higher incidence in the Asian, Native American, and Hispanic populations and lower in the American Black population as compared to the White population. A summary of the New York State program during the years 2000 to 2003 showed some interesting demographic variations in the incidence of congenital hypothyroidism (see Table 1) [5]. As compared to the overall incidence of congenital hypothyroidism, the incidence was somewhat lower in Whites (1:1815) and Blacks (1:1902), somewhat higher in Hispanics (1:1559), and highest in the Asian population (1:1016). In addition, New York found the incidence nearly double in twin births (1:876) as compared to singletons (1:1765), and even higher with multiple births (1:575). Older mothers (> 39 years) had a higher incidence (1:1,328) compared to younger mothers (< 20 years, 1:1,703). The incidence was higher in preterm vs. term infants [5]. It is not clear whether that the congenital hypothyroidism in preterm infants is transient or permanent. However, as the incidence of preterm births has increased by approximately 20 percent over the last 20 years, this may contribute to the reported overall increased incidence. Nearly all screening programs report a female preponderance, approaching 2:1 female to male ratio [6]. A report from Quebec shows this female preponderance occurs mostly with thyroid ectopy, and less so with agenesis [7].

**Table 1:** Incidence of congenital hypothyroidism: Selected demographics from New York State (2000-2003) (modified from: Harris & Pass, *Molec Genet Metab *91:268-277, 2007 [5])

Demographic	Incidence
Overall	1:1681

Gender	

Male	1:1763

Female	1:1601

Ethnicity	

White	1:1815

Black	1:1902

Asian	1:1016

Hispanic	1:1559

Birth weight	

< 1500 g	1:1396

1500 - 2500 g	1:851

> 2500 g	1:1843

Single vs. multiple births	

Single	1:1765

Twin	1:876

Multiple	1:575

Mother's age	

< 20 years	1:1703

20-29 years	1:1608

30-39 years	1:1677

> 39 years	1:1328

## Clinical description

The clinical features of congenital hypothyroidism are often subtle and many newborn infants remain undiagnosed at birth [8,9]. This is due in part to passage of maternal thyroid hormone across the placenta. This is measured in umbilical cord serum to be 25-50 percent of normal [10]. This provides a protective effect, especially to the fetal brain [11]. Also, the most common form of congenital hypothyroidism has some moderately functioning thyroid tissue [12]. The slow development of obvious clinical symptoms [13], coupled with the importance of early treatment led to the implementation of widespread newborn screening for this condition [2]. However, newborn screening for hypothyroidism is not done in many third world countries. Only an estimated 1/3 of the worldwide birth population is screened. It is therefore important that clinicians are able to recognize and treat the disorder.

## Symptoms

Symptoms of congenital hypothyroidism are initially nondescript; however, the maternal and pregnancy history may provide some clues. In twenty percent, gestation extends beyond forty-two weeks [8]. One may also find evidence of maternal autoimmune thyroid disease or an iodine deficient diet. Inadvertent radioactive iodine treatment during pregnancy is rare. Once home, these babies are quiet and may sleep through the night. Additional symptoms include a hoarse cry and constipation. Neonatal hyperbilirubinemia for more than three weeks is common. This is due to immaturity of hepatic glucuronyl transferase [8,14]. Table 2 illustrates some symptoms that were present at the time of diagnosis by newborn screening. In this study, the most common symptoms were prolonged jaundice, lethargy, feeding difficulty and constipation [14].

**Table 2:** Prevalence of individual symptoms of hypothyroidism at the time of diagnosis. (modified from: Alm et al. *Brit Med J *289:1171-175, 1984 [13].)

Features listed in questionnaire	Group 1 (n = 215) Initial T4 ≤ 30 nmol/L % with feature	Group 2 (n = 232) Initial T4 > 30 nmol/L % with feature
Prolonged Jaundice	59	33**
Feeding Difficulty	35	16**
Lethargy	34	14**
Umbilical Hernia	32	18*
Macroglossia	25	12*
Constipation	18	10
Cold or mottled skin	18	10
Hypothermia	3	3
No symptoms	16	33**
Other clinical features reported:		
Abnormal cry	7	6
Edema	5	3
Hypothyroid appearance	6	2
Hypotonia	3	3

**p < 0.001, *p < 0.01.

## Signs

Up to one third have a birth weight greater than the ninetieth percentile [8]. On initial examination, the most common signs are umbilical hernia, macroglossia and cold or mottled skin [14]. Thyroid hormone is also important in the formation and maturation of bone [15,16].This can lead to a wide posterior fontanel of greater than 5 mm. This, along with persistent jaundice and poor feeding are the most striking clinical features [12]. A few infants with congenital hypothyroidism may have a palpable goiter. This is usually found in thyroid dyshormonogenesis where there is a defect in thyroid hormone production. Pendred syndrome mentioned below can present with deafness and a palpable goiter. Other forms of dyshormogenesis are due to defects in enzyme function within the thyroid gland and are discussed further in the section on etiology.

**Figure 1:**
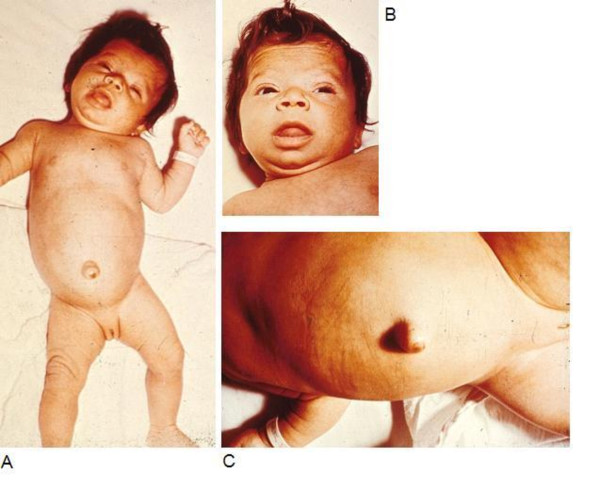
**Infant with congenital hypothyroidism**. A - 3 month old infant with untreated CH; picture demonstrates hypotonic posture, myxedematous facies, macroglossia, and umbilical hernia. B - Same infant, close up of face, showing myxedematous facies, macroglossia, and skin mottling. C - Same infant, close up showing abdominal distension and umbilical hernia.

**Figure 2:**
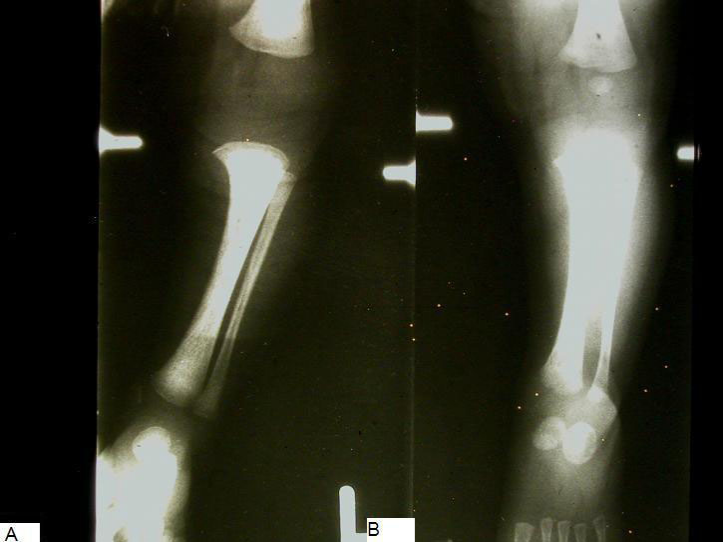
**Radiograph of the left lower extremity of two infants, showing absence of the distal femoral epiphysis on left**. Radiograph of the left lower extremity of two infants. The infant on the left with congenital hypothyroidism demonstrates absence of the distal femoral and proximal tibial epiphyses, while in the normal infant on the right the distal femoral epiphysis is present.

The typical appearance of a hypothyroid infant before the advent of newborn screening is shown in the infant in Figure 1. Features include jaundice, a puffy face and a wide posterior fontanelle with open sutures. The nasal bridge is flat and the eyes exhibit pseudohypertelorism. The mouth may be slightly open revealing macroglossia. Further examination would reveal bradycardia and a protuberant abdomen with a large umbilical hernia. Neurologic examination findings include hypotonia with delayed reflexes. Skin may be cool to touch and mottled in appearance reflecting circulatory compromise [8]. X-rays can reveal absent femoral epiphyses in up to 54% [4]. Figure 2 shows typical radiographs of epiphyseal dysgenesis.

## Congenital malformations

Congenital hypothyroidism appears to be associated with an increased risk of congenital malformations. In one study of 1420 infants with congenital hypothyroidism, extra thyroidal congenital malformations had a prevalence of 8.4%. Of these, the majority were cardiac [17]. Other associated malformations include spiky hair, cleft palate, neurologic abnormalities and genitourinary malformations [17-19]. Also, the incidence of congenital hypothyroidism is increased in patients with Down's Syndrome [20].

**Figure 3:**
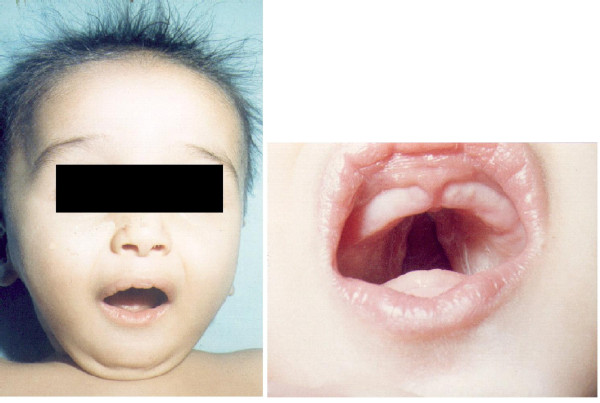
**Bamforth- Lazarus syndrome**. An 8 month old infant with a homozygous mutation in the *TTF*-2 gene locus leading to congenital hypothyroidism. Phenotypic features include, low set ears, extensive cleft palate, hypertelorism, spiky hair and low posterior hairline. (Taken from; A novel loss-of-function mutation in *TTF*-2 is associated with congenital hypothyroidism, thyroid agenesis and cleft palate; Human Molecular Genetics, 2002, Vol. 11, No. 17. Courtesy Dr. Michel Polak and the Oxford University Press.)

Gene mutations causing congenital hypothyroidism can be a rare cause of distinct clinical phenotypes. Most well known is Pendred's syndrome. Affected patients have sensorineural deafness, hypothyroidism and goiter. This syndrome is due to a defect in pendrin, which is a transmembrane chloride-iodide transporter expressed in both the thyroid gland and the inner ear [21]. A mutation in thyroid transcription factor 2 (*TTF-2*) causes a syndrome of thyroid dysgenesis, choanal atresia, cleft palate and spiky hair also known as Bamforth-Lazarus syndrome [22] (Figure 3). Mutations in *NKX 2.1 *causes congenital hypothyroidism associated with respiratory distress and neurologic problems such as benign hereditary chorea and ataxia [23-25].

One clinical manifestation of long standing congenital hypothyroidism is the Kocher-Debre- Semelaigne syndrome. This presents as promixal muscle weakness associated with calf hypertrophy and resolves with thyroid hormone treatment [26]. Other clinical syndromes that include congenital hypothyroidism are included under "Syndromic hypothyroidism" in Table 3.

## Etiology

Permanent congenital hypothyroidism may be due to primary or secondary (central) causes. Primary causes include defects of thyroid gland development, deficiencies in thyroid hormone production, and hypothyroidism resulting from defects of TSH binding or signal transduction. Peripheral hypothyroidism results from defects in thyroid hormone transport, metabolism, or resistance to thyroid hormone action. Secondary or central causes include defects of thyrotropin releasing hormone (TRH) formation or binding and TSH production. These are covered briefly in this review and are listed in Table 3.

**Table 3:** Classification and etiology of congenital hypothyroidism

**1**.	Primary hypothyroidism
	Thyroid dysgenesis: hypothyroidism due to a developmental anomaly
	(Thyroid ectopia, athyreosis, hypoplasia, hemiagenesis)
	Associated mutations: (these account for only 2% of thyroid dysgenesis cases; 98% unknown)
	TTF-2,
	NKX2.1,
	NKX2.5
	PAX-9
	Thyroid dyshormonogenesis: hypothyroidism due to impaired hormone production
	Associated mutations:
	Sodium-iodide symporter defect
	Thyroid peroxidase defects
	Hydrogen peroxide generation defects (DUOX2, DUOXA2 gene mutations)
	Pendrin defect (Pendred syndrome)
	Thyroglobulin defect
	Iodotyrosine deiododinase defect (DEHAL1, SECISBP2 gene mutations)
	Resistance to TSH binding or signaling
	Associated mutations:
	TSH receptor defect
	G-protein mutation: pseudohypoparathyroidism type 1a

**2**.	**Central hypothyroidism (syn: Secondary hypothyroidism)**

	Isolated TSH deficiency (TSH β subunit gene mutation)
	Thyrotropin-releasing hormone deficiency
	Isolated, pituitary stalk interruption syndrome (PSIS), hypothalamic lesion, e.g. hamartoma
	Thyrotropin-releasing hormone resistance
	TRH receptor gene mutation
	Hypothyroidism due to deficient transcription factors involved in pituitary development or function
	HESX1, LHX3, LHX4, PIT1, PROP1 gene mutations

**3**.	**Peripheral hypothyroidism**

	Resistance to thyroid hormone
	Thyroid receptor β mutation
	Abnormalities of thyroid hormone transport
	Allan-Herndon-Dudley syndrome (monocarboxylase transporter 8 [MCT8] gene mutation)

**4**.	**Syndromic hypothyroidism**

	Pendred syndrome - (hypothyroidism- deafness - goiter) **Pendrin mutation**
	Bamforth-Lazarus syndrome - (hypothyroidism - cleft palate - spiky hair) **TTF-2 mutation**
	Ectodermal dysplasia - (hypohidrotic - hypothyroidism - ciliary dyskinesia)
	Hypothyroidism - (dysmorphism - postaxial polydactyly - intellectual deficit)
	Kocher - Deber - Semilange syndrome - (muscular pseudohypertrophy- hypothyroidism)
	Benign chorea - hypothyroidism
	Choreoathetosis - (hypothyroidism - neonatal respiratory distress) **NKX2.1 /TTF-1 mutation**
	Obesity - colitis - (hypothyroidism - cardiac hypertrophy - developmental delay)

**5**.	**Transient congenital hypothyroidism**

	Maternal intake of antithyroid drugs
	Transplacental passage of maternal TSH receptor blocking antibodies
	Maternal and neonatal iodine deficiency or excess
	Heterozygous mutations of THOX2 or DUOXA2
	Congenital hepatic hemangioma/hemangioendothelioma

Transient hypothyroidism may be caused by maternal or neonatal factors. Maternal factors include antithyroid medications, transplacental thyrotropin receptor blocking antibodies and exposure to iodine deficiency or excess. Neonatal factors include, neonatal iodine deficiency or excess, congenital liver hemangiomas and mutations in the genes encoding for *DUOX *and *DUOXA2 *(see Table 3).

## Permanent congenital hypothyroidism

In iodine sufficient countries, 85% of congenital hypothyroidism is due to thyroid dysgenesis. This term refers to an aberration of the embryological development of the thyroid gland. The remaining 10-15% of cases can be attributed to the inborn errors of thyroid hormone synthesis, also called dyshormonogenesis, or to defects in peripheral thyroid hormone transport, metabolism, or action [27].

## Thyroid dysgenesis

Thyroid dysgenesis presents in three major forms: thyroid ectopy, athyreosis and thyroid hypoplasia. Thyroid ectopy refers to an ectopic location of the thyroid gland. This accounts for two-thirds of congenital hypothyroidism due to thyroid dysgenesis and is twice as common in females [28]. In these cases, a thyroid remnant is usually found along the normal pathway of the thyroglossal duct. This represents the path taken by the developing thyroid as it descends from the base of the tongue to its final location in the neck [28,29]. In one study done on hypothyroid neonates, ectopic thyroid tissue was found inferior and superior to the hyoid bone, and above the thyroid cartilage [30]. Athyreosis refers to the complete absence of thyroid tissue. Athyreosis and thyroid hypoplasia account for the remaining one third of thyroid dysgenesis.

Table 4 illustrates the relative frequencies of the different forms of congenital hypothyroidism found in patients screened in the Quebec newborn screening program from 1990-2004.

**Table 4:** Etiology of congenital hypothyroidism in 148 patients diagnosed in the Quebec Newborn Screening program from 1990-2004. (modified from: Eugene et al. *J Clin Endocrinol Metab 90:2696-2700*, 2005 [111])

	Female	Male	Total	Percentage
Athyreosis	14	10	24	16

Ectopic	78	24	102	68

Orthotopic/dyshormonogenesis	9	13	22	15

Totals	101	47	148	100

Thyroid dysgenesis is generally thought to be sporadic in occurrence. However, recent evidence points to the possibility of a genetic component. One study of all cases of thyroid dysgenesis found that 2% were familial in occurrence [31]. Additional studies also showed that 7.9% of first degree relatives of infants with congenital hypothyroidism had a thyroid developmental anomaly [32]. There was some speculation as to a possible seasonal variation in the incidence of congenital hypothyroidism; however, this topic is still under debate [33,34].

Some genes have been implicated as a cause of thyroid dysgenesis. However, these generally account for a small number of cases [35]. These include paired box gene eight (*PAX8*), *TTF-2*, *NKX2.1 *and *NXK2.5 *[22-25][36-39]. These encode for transcription factors which are expressed both during thyroid embryogenesis and in the normal functioning gland [29]. These transcription factors are also expressed in other tissues of the developing fetus. Mutations in genes coding for these transcription factors lead to distinct phenotypic syndromes which are linked to their tissue expression [40]. These are described below (see Table 5).

**Table 5:** Transcription factor gene mutations resulting in thyroid dysgenesis and associated clinical findings

Mutated Gene	Associated clinical findings
Thyroid transcription factor 2 (*TTF2*)	thyroid dysgenesis, choanal atresia, cleft palate and spiky hair

*NKX2.1*	congenital hypothyroidism, respiratory distress ataxia and benign chorea

*NKX2.5*	Congenital hypothyroidism and cardiac malformations

*PAX-8*	Thyroid dysgenesis, kidney and ureteral malformations

• *TTF-2 *- a homozygous missense mutation in *TTF-2 *causes a genetic syndrome of thyroid dysgenesis, choanal atresia, cleft palate and spiky hair [22]. This syndrome has been recently referred to as Bamforth-Lazarus Syndrome [35].

• *NKX2.1 *- mutations in *NKX2.1*, also known as *TTF-1*, have been associated with congenital hypothyroidism, respiratory distress and ataxia [23,24]. Also, recent reports describe an *NKX2.1 *mutation with congenital hypothyroidism and benign chorea [25,38].

• *NKX2.5 *has been expressed in cardiac tissues; the recent finding of *NKX2.5 *mutations in patients with thyroid dysgenesis suggest a genetic cause for the increased incidence of cardiac malformations in congenital hypothyroidism[39].

In contrast, *PAX8 *mutations seem to cause thyroid dysgenesis in the absence of other congenital anomalies [35-37]. However, given that *PAX8 *is also expressed in the mesonephros and ureteric buds [40], this may explain the increased incidence of genitourinary malformations in patients with congenital hypothyroidism [19].

## TSH resistance

There are several forms of TSH resistance. Mutations in the TSH receptor gene leading to thyroid hypoplasia have been found [41]. Another form of TSH resistance is dominantly inherited and has been linked to the long arm of chromosome 15 [42]. Resistance occurs in the absence of a TSH receptor mutation and can again cause thyroid hypoplasia [43]. Pseudohypoparathyroidism type 1a, caused by mutations in the alpha subunit of the stimulatory guanine nucleotide binding protein (Gs alpha), results in defective TSH signaling [44].

## Thyroid dyshormonogenesis

Hereditary defects in virtually all the steps of thyroid hormone biosynthesis and secretions have been described and account for 10-15% of permanent congenital hypothyroidism. These are generally transmitted in an autosomal recessive manner, but at least one condition has autosomal dominant inheritance. Dyshormonogenesis leads to goitrous hypothyroidism; however, this is rarely seen in babies detected by newborn screening [45].

Most commonly, dyshormonogenesis is due to defects of thyroid peroxidase activity [46]. Thyroid peroxidase uses hydrogen peroxide to couple iodine to thyroglobulin within the thyroid gland, forming T3 and T4. Severe defects in this enzyme lead to total iodide organification defects (TIOD). This diagnosis is made by showing high radioactive iodine (RAI) uptake of the thyroid gland followed by more than 90% release after sodium perchlorate administration [47] (see section on diagnosis). Less severe mutations cause partial iodide organification defects (PIOD). Pendred's syndrome is a well known form of syndromic hypothyroidism and is characterized by a triad of hypothyroidism, goiter and deafness. This syndrome is caused by a genetic defect in the transmembrane protein pendrin (encoded on 7q31), which acts as a chloride-iodide transporter in both in the thyroid gland and the inner ear. Defects in pendrin lead to impaired iodide organification and these patients have a positive perchlorate discharge test [21]. More recently, mutations in the enzyme dual oxidase 2 (known as *DUOX2 *or *THOX2*) have been found. They lead to dyshormonogenesis from deficient hydrogen peroxide generation and can be autosomal dominant. Their phenotype is heterogeneous and can be permanent or transient and cause either total or partial iodide organification defects [48]. Mutations in the dual oxidase maturation factor (*DUOXA2*) gene also lead to deficient iodide organification through similar mechanisms and can cause partial iodide organification defects [49]. Other, rare causes of dyshormonogenesis include defects in sodium/iodide transport, resulting from a mutation in the gene encoding the sodium-iodide symporter [50], and defective thyroglobulin action, resulting from a mutation in the gene encoding thyroglobulin [51]. A defect in the enzyme iodotyrosine deiodinase which aids in the peripheral conversion of T4 to T3 has been shown in hypothyroid individuals. This can be due to homozygous mutations in the genes *DEHAL1 *or *SECISBP2 *[52,53].

## Secondary or Central hypothyroidism

Congenital secondary or central hypothyroidism generally results from defects of TSH production; most commonly, it is part of a disorder causing congenital hypopituitarism. Congenital hypopituitarism often is associated with midline defects such as septo-optic dysplasia or cleft lip and/or palate and can be part of a larger genetic syndrome. Mutations in genes regulating pituitary gland development, which include HESX1, LHX3, LHX4, PIT1 and PROP1 have been reported to be a cause of familial hypopituitarism. Besides TSH deficiency, other pituitary hormones are often deficient, including growth hormone, adrenocorticotrophic hormone and antidiuretic hormone. Rarely, specific gene defects lead to central hypothyroidism. These include isolated TSH deficiency (autosomal recessive, caused by mutations in the TSH β subunit gene), and thyrotropin releasing hormone (TRH) resistance, resulting from mutations in the TRH receptor gene (see Table 3).

## Peripheral defects in thyroid hormone metabolism

Passage of thyroid hormone into cells is facilitated by thyroid hormone plasma membrane transporters. A mutation in a gene encoding monocarboxylase transporter 8 (*MCT8*) has been reported in five boys as a cause of X-linked hypothyroidism associated with mental retardation and neurologic abnormalities including quadriplegia. The defective transporter appears to impair the passage of T3 into neurons and is characterized by elevated serum T3 levels, low T4 and normal TSH [54]. This is also known as Allan-Herndon-Dudley syndrome.

Peripheral resistance to the action of thyroid hormone has been described. This is due in 90% of cases to mutations in genes encoding for thyroid hormone receptor β (TR β). These mutations are dominantly inherited and affected individuals are generally euthyroid, however some hypothyroid individuals have been described. Circulating T3 and T4 are mildly elevated without suppression of TSH. Thus these infants are usually not detected by newborn screening [55].

## Transient congenital hypothyroidism

Transient congenital hypothyroidism is found to be more common in Europe (1:100) than the United States (1:50,000) [3]. In a report of over twenty years in the French newborn screening program, the incidence of transient congenital hypothyroidism was found to be 40 percent [3]. Causes of transient congenital hypothyroidism include:

• Iodine deficiency - Iodine deficiency is more common in European countries, especially in preterm infants; this is due mainly to maternal iodine deficient diets [3,12,56].

• Transfer of maternal blocking antibodies - Maternal antithyroid antibodies can cross the placenta and block the TSH receptor in the neonatal thyroid. This effect can last up to 3 to 6 months after birth as maternal antibody levels fall [57,58].

• Fetal exposure to antithyroid drugs - Maternal antithyroid drugs can cause decreased neonatal thyroid hormone synthesis which lasts for a few days to two weeks after birth.

• Maternal iodine exposure - Maternally administered amiodarone may cause transient hypothyroidism in their infants. This seems to resolve at around 4-5 months of age and can be associated with adverse neurologic outcomes [59]. Transient hypothyroidism also occurs when iodine antiseptic compounds are used on mothers or after exposure to iodinated contrast agents; however, this may be related to the type and duration of exposure as a recent study showed no abnormal thyroid functions in the infants of 21 mothers given iodide contrast during pregnancy [60].

• Neonatal Iodine exposure - Exposure of newborns to high amounts of iodine can cause hypothyroidism. This can occur especially in preterm infants[61].

• Liver hemangiomas - There are reports of congenital liver hemangiomas that produce large amounts of the enzyme type 3 iodothyronine deiodinase. This produces a consumptive type of hypothyroidism in which large doses of thyroxine are required to maintain euthyroidism. Serum T4 levels are low, TSH is elevated, and reverse T3 levels are also increased. Hypothyrodism resolves as the tumor involutes or is treated [62].

• Mutations in *DUOX2 *(*THOX2*) and *DUOXA2 *can lead to transient congenital hypothyroidism as previously described [48,49].

## Diagnosis

In those countries with newborn screening programs in place, essentially all infants with congenital hypothyroidism are diagnosed after detection by newborn screening tests. Screening programs have been developed in Canada, the United States, parts of Mexico, Western Europe, Japan, Australia, New Zealand, and Israel, and they are under development in parts of many countries in Eastern Europe, Asia, South America and Africa. Of the worldwide birth population of 127 million, it is estimated that 25 percent undergo screening for congenital hypothyroidism. In infants born in locations without newborn screening programs, diagnosis may be made after development of clinical manifestations of hypothyroidism.

## Newborn thyroid screening tests

**Figure 4:**
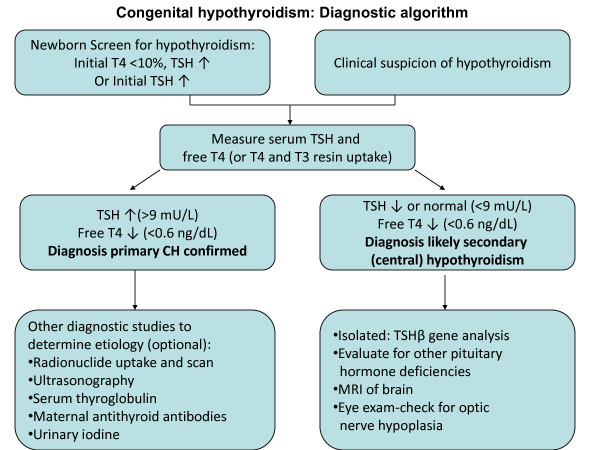
**Congenital hypothyroidism: Diagnostic algorithm**. Diagnostic algorithm: the diagnosis of congenital hypothyroidism begins with either abnormal newborn screening test results or a clinical suspicion of hypothyroidism, leading to serum thyroid function tests (typically TSH and free T4) to confirm the diagnosis. If a diagnosis of primary or secondary (central) congenital hypothyroidism is confirmed, other diagnostic studies can be undertaken to determine the underlying etiology.

The specimen used for newborn screening tests is blood from a heel-prick collected on special filter paper cards. The specimen is routinely collected between two and five days of age (or at discharge from the hospital, if this occurs earlier); some programs use cord blood for screening. In addition, some programs also routinely obtain a 2 ^nd ^specimen between two and six weeks of age. The filter paper cards are then sent to a centralized laboratory for testing. Early in the experience of screening, most programs undertook an initial T4 test, with a follow-up TSH test on infants below a specified T4 cutoff [2]. With increasing accuracy of TSH measurement, many screening programs now carry out an initial TSH test to detect congenital hypothyroidism. Each program must develop its own T4 and TSH cutoff for recall of infants with abnormal test results (see Figure 4, Diagnostic algorithm). As there are rapid changes in TSH and T4 in the first few days of life, many programs have developed age-related cutoffs. Following are examples of typical cutoffs for T4 and TSH:

• Initial T4 < 10 ^th ^percentile →follow-up TSH test

• Initial TSH > 30 mU/L serum (> 15 mU/L whole blood)); some programs use an upper percentile TSH cutoff, e.g., > 97 ^th ^percentile

Both of the above screening test approaches will detect the majority of infants with primary congenital hypothyroidism. There are some advantages and disadvantages with each approach in the detection of other thyroid disorders. Both screening test approaches do a good job of detecting infants with primary CH. The primary T4-follow-up TSH test strategy will detect some infants with secondary or central (hypopituitary) hypothyroidism and infants with "delayed TSH rise". On the other hand, a primary TSH test strategy will detect infants with mild or "subclinical" hypothyroidism (see Table 6). Neither test approach will detect infants with defects of thyroid transport, metabolism, or action. With a goal of detecting all of these thyroid disorders, some programs have undertaken pilot programs measuring both T4 and TSH on all newborns. These programs tend to report a higher incidence of congenital hypothyroidism [63].

**Table 6:** A comparison of the thyroid disorders detected by primary T4-follow-up TSH testing vs. primary TSH testing

Disorder	Primary T4-Follow-up TSH	Primary TSH
Primary CH	Good	Good

Central CH	Some	No

Mild CH	No	Yes

Delayed rise TSH	Yes	No

Unusual forms of CH, e.g., defects of thyroid cell membrane transport (MCT8), metabolism or action	No	No

## Confirmatory serum thyroid testing

Once an infant has been detected with abnormal thyroid screening tests, they should be recalled immediately for examination, and a venapunctue blood sample should be obtained for confirmatory serum testing (see Figure 4, Diagnostic algorithm). Confirmatory serum is tested for TSH and either free T4 or total T4 combined with some measure of binding proteins, such as a T3 resin uptake.

It is important to compare the serum results with age-normal reference ranges. In the first few days of life, serum TSH can be as high as 39 mU/L, as a result of the TSH surge that occurs shortly after birth (this is the reason that the filter paper screening test cutoff is approximately 30 mU/L). Most confirmatory serum tests are obtained around one to two weeks of life, when the upper TSH range falls to approximately 10 mU/L. The approximate normal reference ranges for serum free T4, total T4, and TSH in the first 4 weeks of life are shown in Table 7. Although levels of all hormones are higher at 1-4 days of age, by 2-4 weeks of age they have fallen closer to the levels typically seen in infancy.

**Table 7:** Reference ranges for thyroid function tests at ages 1-4 days and 2-4 weeks [64]

Age	Free T4 (pmol/L)	Total T4 (nmol/L)	TSH (mU/L)
1-4 days	25-64	129-283	< 39

2-4 weeks	10-26	90-206	< 10

The finding of an elevated serum TSH level and a low free T4 or total T4 confirms the diagnosis of primary hypothyroidism. The finding of an elevated serum TSH with a normal free T4 or total T4 is consistent with subclinical primary hypothyroidism. Because of the dependence of the developing brain on optimal concentrations of thyroid hormone, we recommend treating infants with subclinical hypothyroidism.

It is now recognized that preterm infants or acutely ill term infants with primary hypothyroidism may not show an elevated TSH level on the 1 ^st ^screening test. Thus, many programs undertake a routine 2 ^nd ^screening test in preterm and acutely ill term infants. Such testing leads to the detection of infants with "delayed TSH rise", which occurs in approximately 1:18,000 newborns [64].

Those programs that undertake a primary T4 test and recall infants with persistently low T4 screening levels, e.g. on the routine 1 ^st ^and 2 ^nd ^specimens (without elevated TSH levels) detect some infants with secondary or central hypothyroidism [65]. Confirmatory serum testing will show a low free T4 or total T4, with either a low TSH or an "inappropriately normal" TSH level. In infants with central hypothyroidism, the TSH deficiency may be isolated, as in infants with TSHβ gene mutations. In most cases of central hypothyroidism, however, TSH deficiency is associated with other pituitary hormone deficiencies. Such infant should be investigated for other pituitary hormone deficiencies, particularly if they have features such as hypoglycemia, which is suggestive of growth hormone (GH) and/or ACTH deficiency, or micropenis and undescended testes in a male, which are suggestive of gonadotropin (LH, FSH) deficiencies.

Some infants who undergo serum testing because of "low T4, non-elevated TSH" screening test results will be discovered to have thyroxine binding globulin (TBG) deficiency. Serum testing will show a low total T4, but a normal free T4 and normal TSH level. TBG deficiency can be confirmed by finding a low serum TBG level. TBG deficiency is an X-linked recessive disorder that occurs in approximately 1:4,000 infants, primarily males [66]. These infants are euthyroid and treatment is not necessary.

## Diagnostic studies to determine an underlying etiology

Treatment of congenital hypothyroidism is based on serum thyroid function test results, as outlined above. Other diagnostic studies may be undertaken to determine an underlying etiology. Such diagnostic studies may include thyroid radionuclide uptake and scan, thyroid ultrasonography, serum thyroglobulin (Tg) measurement, antithyroid antibody determinations, and measurement of urinary iodine (see Table 8). Findings may guide treatment decisions in infants with borderline serum test results, e.g., discovery of a form of thyroid dysgenesis. Results from these tests will usually separate transient from permanent cases. If a familial form of congenital hypothyroidism is discovered, this will guide genetic counseling. As gene mutations have now been reported for virtually all steps in thyroid hormone synthesis, the above diagnostic studies may lead to a specific genetic test to confirm the underlying etiology. In addition, these diagnostic studies may be performed routinely in programs that use this information for clinical investigations. However, these diagnostic studies generally do not alter the treatment decision, and so they are considered optional.

**Table 8:** Findings in diagnostic studies undertaken to identify the underlying etiology of congenital hypothyroidism

Defect	Radionuclide image	Ultrasonography	Serum thyroglobulin	Maternal TRB-Ab
Aplasia	No uptake	Absent gland	Low	Negative

Hypoplasia	↓ uptake	Small, eutopic	Intermediate	Negative

Ectopia	↓ uptake, ectopic	Ectopic gland (hypoplastic)	Intermediate	Negative

TSHβ mutations	No uptake	Eutopic gland (hypoplastic)	Intermediate	Negative

TSH receptor inactivating mutation	↓ uptake	Eutopic gland	Intermediate-high	Negative

Trapping error	↓ or no uptake	Eutopic gland	Low-intermediate	Negative

Beyond trapping error	↑ uptake	Eutopic, large gland	High Exception: Tg gene mutations	Negative

Maternal TRB-Ab	↓ or no uptake	Eutopic gland	Low-intermediate	Positive

## 1. Thyroid radionuclide uptake and scan

**Figure 5:**
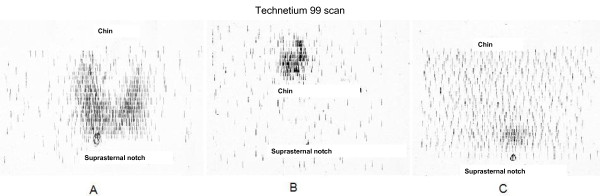
**Technetium 99 m scan findings in congenital hypothyroidism**. A-Technetium 99 m scan, showing a large gland (approximately twice normal size) in eutopic location, consistent with dyshormonogenesis. B-Technetium 99 m scan, showing uptake in ectopic location, i.e. ectopic gland. C-Minimal uptake, consistent with aplasia or severe hypoplasia.

Either iodine-123 (I-123) or sodium pertechnetate 99 m (Tc99 m) are preferred for thyroid uptake and scan in neonates to minimize the radioactivity exposure; I-131 delivers a higher dose to the thyroid and total body and should not be used. Radionuclide uptake and scanning generally are the most accurate tests in defining some form of thyroid dysgenesis, e.g., an ectopic gland, thyroid hypoplasia (decreased uptake in a eutopic location), or thyroid aplasia [67] (Figure 5). Absence of radionuclide uptake should be confirmed by an ultrasonography. Absence of uptake can also be seen with TSHβ gene mutations, TSH receptor inactivating mutations, iodide trapping defects, and with maternal thyrotropin receptor blocking antibodies (TRB-Ab); thyroid ultrasonography and other studies, such as measurement of serum Tg or TRB-Ab will help to separate these etiologies from thyroid aplasia (see below).

If radionuclide studies show a large gland in a eutopic location with increased uptake, these findings are suggestive of one of the dyshormonogeneses beyond trapping. In such cases, I-123 uptake can be followed by a perchlorate discharge test. If there is defective oxidation and organification of iodide, it will not be attached to tyrosine on thyroglobulin, and so it will be rapidly "discharged" from the thyroid gland when high doses of perchlorate are given. Genetic studies for a mutation in thyroid peroxidase, the enzyme responsible for oxidation and organification, can confirm this inborn error of thyroid hormone biosynthesis.

## 2. Thyroid ultrasonography

Thyroid ultrasonography is accurate in confirming true thyroid aplasia. In the situations described above where radionuclide uptake and scan show absent uptake, but a gland is actually present (TSHβ gene mutations, TSH receptor inactivating mutation, iodide trapping defect, maternal TRB-Ab), ultrasonography may show a thyroid gland in a eutopic location. Ultrasonography generally is not as accurate as radionuclide scan in demonstrating ectopic glands [68]. More recent studies report that color flow doppler ultrasonography is able to detect ectopic thyroid tissue in 90 percent of infants with ectopic glands detected by radionuclide imaging [69]. Thyroid ultrasonography can confirm a large gland, suggestive of dyshormonogenesis.

## 3. Serum thyroglobulin (Tg) determination

Serum thyroglobulin levels reflect the amount of thyroid tissue and generally are elevated with increased thyroid activity, as when TSH is elevated. In addition, with inflammation, more thyroglobulin "leaks" into the circulation. One study showed that serum thyroglobulin levels were lowest in neonates with thyroid aplasia (mean 12 ng/mL, range 2 to 54 ng/mL), intermediate with ectopic glands (mean 92 ng/mL, range 11 to 231 ng/mL), and highest in cases associated with large glands (mean 226 ng/mL, range 3 to 425 ng/mL) [68]. Thus, while these groups could be separated by their serum thyroglobulin levels, given the degree of overlap it could not be used to diagnose the etiology in individual cases. In cases of true thyroid aplasia, serum thyroglobulin levels are absent if measured a few weeks after birth. Serum thyroglobulin determinations can be useful in cases of absent radionuclide uptake. If the thyroglobulin level is increased, this suggests that the thyroid gland is present, and that the neonate may have a TSH receptor inactivating mutation [70], a trapping defect, or maternal TRB-Ab, rather than aplasia.

## 4. Anti-thyroid antibodies

Maternal autoimmune thyroid disease may be associated with production of a thryotropin receptor blocking antibody (TRB-Ab). This antibody will cross to the fetus and block TSH binding, inhibiting fetal thyroid gland development and function. Maternal autoimmune thyroid disease is relatively common, as approximately 5 percent of women of reproductive age have either anti-thyroglobulin or thyroid peroxidase antibodies [71]. However, maternal TRB-Ab is relatively rare, causing transient congenital hypothyroidism in approximately1:100,000 neonates [57]. Thus, we only recommend TRB-Ab determinations in a case where a previous child has had transient congenital hypothyroidism, and mother has known autoimmune thyroid disease and is pregnant again. TRB-Ab can be screened for by a thyrotropin binding inhibitor immunoglobulin (TBII) determination in mother.

## 5. Urinary iodine determination

If an infant with congenital hypothyroidism is born in an area of endemic iodine deficiency, or if there is a history of excess iodine exposure, measurement of urinary iodine may confirm either iodine deficiency or excess. 24 hour urinary iodine approximates iodine intake; the normal range in neonates is approximately 50 to 100 mcg.

## 6. Genetic mutations

Testing for specific genetic mutations generally is only considered after other studies point to a specific defect, e.g, a mutation in the gene for thyroid peroxidase in a neonate with a goiter, increased radionuclide uptake, and positive perchlorate discharge test. As noted previously, mutations in *TTF-1*, *NKX2.1*, or *PAX-8 *genes are found in only 2% of cases of thyroid dysgenesis. Thus, in the vast majority of cases of thyroid dysgenesis, the underlying cause remains unknown.

Laboratories around the world offer genetic testing for most of the following genetic disorders[72,73]:

• TSHβ mutations

• TSH receptor inactivating mutations

• Thyroid dysgenesis

◦ *TTF-2 *mutations

◦ *NKX2.1 *mutations

◦ *PAX-8 *mutations

• Thyroid dyshormonegenesis

◦ Sodium-iodide symporter mutations

◦ Hydrogen peroxide mutations

▪ *DUOX2 *mutations

▪ *DUOX2A *mutations

◦ Thyroid peroxidase mutations

▪ Pendred syndrome (PDS): pendrin gene mutations

◦ Thyroglobulin mutations

◦ Deiodinase mutations

• Defects in thyroid hormone transport

◦ MCT8 mutation

## Differential diagnosis

In cases where an infant with congenital hypothyroidism is detected by newborn screening tests and the diagnosis is confirmed by serum thyroid function tests, a clinical differential diagnosis is not considered. As described under **confirmatory serum thyroid testing **(above), results will lead to a diagnosis of primary congenital hypothyroidism, subclinical hypothyroidism, and, in some programs, secondary or central hypothyroidism.

In the absence of newborn screening programs, the diagnosis of congenital hypothyroidism is made after development of clinical manifestations. Since symptoms and signs develop gradually after birth, the diagnosis of hypothyroidism may be difficult at first. The timing of clinical features will vary depending on the severity of hypothyroidism. The myxedematous facial features, flat nasal bridge, macroglossia, and hypotonia may suggest Down syndrome or a metabolic storage disease. Prolonged jaundice and a protuberant abdomen may suggest a congenital liver disorder such as biliary atresia. Slow linear growth, a large head with immature body proportions, and radiological features of epiphyseal dysgenesis may be mistaken for a skeletal dysplasia or pituitary dwarfism. Eventually the clinical symptoms and signs suggest congenital hypothyroidism, and appropriate thyroid function tests confirm the diagnosis.

## Genetic counseling

The most common cause of congenital hypothyroidism, thyroid dysgenesis, is typically a sporadic disorder, and so there is no recurrence risk with future pregnancies. The sporadic nature is supported by twin studies, which show a discordance for thyroid dysgenesis in both monozygotic and dizygotic twins [74]. There is evidence of a familial component in some cases of thyroid dysgenesis (aplasia, hypoplasia and ectopic glands). In a report of 2,472 patients with congenital hypothyroidism identified by the French newborn screening program and shown to be a result of thyroid dysgenesis, 48 (2 percent) appeared to be familial (typically occurring in siblings or cousins, but also some mother/father-daughter/sons) [28].Further evidence for a familial component comes from a French study which reported that 21.4 percent of first degree relatives of patients with congenital hypothyroidism had asymptomatic thyroid developmental anomalies, such as thyroglossal duct cysts, hemiagenesis, or a pyramidal lobe, as compared to 0.9 percent in a control population [32]. This study suggests a common genetic component between thyroid dysgenesis and these developmental anomalies. Rare cases of apparent thyroid agenesis have been reported in patients with loss-of-function mutations of the TSH receptor [70]. These cases appear to have an autosomal recessive pattern of inheritance. In summary, if a patient is detected with congenital hypothyroidism and imaging studies show some form of thyroid dysgenesis, the families can be counseled that the recurrence risk appears small, around 2 percent.

A minority of patients develop congenital hypothyroidism as a result of a hereditary defect in thyroid hormone biosynthesis, one of the dyshormonogeneses. Dyshormonogenesis may be suspected in an infant detected with congenital hypothyroidism and a goiter. These inborn errors of thyroid hormone biosysnthesis are the result of mutations in the sodium-iodide symporter, thyroid peroxidase, thyroglobulin, or iodotyrosine deiodinase genes. All of these inborn errors are autosomal recessive, and so they carry a 25 percent recurrence risk in future pregnancies. One specific disorder, Pendred's syndrome consists of sensorineural deafness, goiter, and impaired iodide organification. Pendred's syndrome is also an autosomal recessive disorder, linked to chromosome 7q22-33.1, and results from a mutation in a chloride-iodide transport protein expressed in the thyroid and inner ear [21]. While some patients with Pendred syndrome may develop hypothyroidism at birth [75], the majority are clinically and biochemically euthyroid.

There is a high recurrence risk of hypothyroidism in babies born to mothers with autoimmune thyroid disease associated with a thyrotropin receptor blocking antibody (TRB-Ab). Mothers should be investigated for TRB-Ab in cases of recurrent congenital hypothyroidism in siblings. The TRB-Ab will cross the placenta and block fetal thyroid gland development. Although this is an uncommon cause of congenital hypothyroidism [57], mothers should be counseled that as long as they have a high concentration of TRB-Ab, future pregnancies are at risk.

## Antenatal diagnosis

As congenital hypothyroidism is most commonly not a heritable disorder, and the majority of cases are sporadic, it is not possible to identify a population of pregnant women who are at high risk for fetal hypothyroidism. Specific pregnancies may be determined to be at risk based on a family history of a previous infant with congenital hypothyroidism, for example resulting from dyshormonogenesis or maternal TRB-Ab. Most cases, however, are not familial and are discovered when routine ultrasonography discloses a fetal goiter [76]. In addition, if a pregnant woman with Graves' disease is treated with antithyroid drugs, the fetus is at risk for hypothyroidism. Further, if a pregnant woman inadvertently receives radioactive iodine (RAI) after 8-10 weeks gestation, the fetal thyroid will trap the RAI, resulting in thyroid ablation and hypothyroidism.

There are rare case reports of subsequent pregnancies in families where a previous sibling had a familial form of congenital hypothyroidism [77]. In cases where a fetus is at risk for hypothyroidism, e.g., a previous infant with congenital hypothyroidism caused by dyshormonogenesis, or one of the rarer forms of familial thyroid dysgenesis, or a defect in thyroid hormone transport, where a genetic defect has been identified, genetic testing on fetal cells obtained by amniocentesis will determine whether or not the current pregnancy is also affected (for a list of defects, see 6. Genetic mutations, under "Diagnosis"). In these recessive disorders, with a recurrence risk of 25 percent, subsequent affected pregnancies may be suspected based on ultrasound findings of a fetal goiter, along with increased amniotic fluid and fetal bradycardia. In general, measurement of amniotic fluid TSH or thyroid hormone levels are not reliable, and sampling of fetal umbilical cord blood is necessary to diagnose fetal hypothyroidism. One case discovered by routine antenatal ultrasonography reported simultaneous amniotic fluid and fetal cord TSH measurements [78]. At 32 weeks gestation, amniotic fluid TSH was 8.76 mU/L while fetal cord blood TSH was 231.00 mU/L. With intra-amniotic injections of levothyroxine (l-thyroxine), the amniotic fluid TSH fell to 0.95 mU/L, while the cord blood TSH fell to 1.20 mU/L. The authors felt in this case that the initial amniotic fluid TSH was elevated (normal range 0.15-1.7 mU/L) and diagnostic of fetal hypothyroidism [78]. In general, genetic testing on fetal cells obtained by amniocentesis is a more direct and safer method of diagnosis than fetal cord blood sampling.

Several cases of hypothyroidism diagnosed antenatally have undergone treatment via intra-amniotic injections of l-thyroxine [79]. Typically, 250 mcg of l-thyroxine (range 250 to 600 mcg) has been injected weekly into the amniotic fluid. Subsequent dosing was based on the treatment effect in reducing the size of the fetal goiter and on repeat fetal cord blood thyroid tests. In general, such antenatal monitoring and treatment is well tolerated, although the risks of amniotic fluid injections and fetal cord blood sampling include premature labor, bleeding, and infection. While most cases report good psychomotor developmental outcome, there have not been any systematic studies of antenatal treatment of fetal hypothyroidism. Considering that the majority of infants with congenital hypothyroidism do well if detected by newborn screening with treatment started within the first 2-4 weeks of life, it is unclear if antenatal treatment is necessary for optimal neurocognitive outcome, though it clearly is successful in shrinking fetal goiter.

## The management of congenital hypothyroidism

Congenital hypothyroidism is one of the most common treatable causes of mental retardation. Studies have shown that the timing of therapy is crucial to neurologic outcome. Indeed, there is an inverse relationship between intelligence quotient (IQ) and the age at diagnosis [13,80]. Even when diagnosed early, neurologic development may suffer if treatment is not optimized in the first two to three years of life [81]. It is therefore important for these patients to receive early treatment and close follow up.

The overall goal of therapy is ensure that these patients are able to have growth and mental development that is as close as possible to their genetic potential. This is achieved by rapidly restoring the free T4 and the TSH to the normal range and then maintaining clinical and biochemical euthyroidism.

## Formulations

Levothyroxine (l-thyroxine) is the treatment of choice. Although triiodithyronine (T3) is the biologically active form of the hormone, most T3 in the brain is formed from local deiodination of T4; thus, T3 replacement is not needed for normal neurologic functioning. In a study of forty seven infants given varying treatment doses of l-thyroxine, serum T3 normalized and remained normal regardless of the treatment dose used, again suggesting that treatment with l-thyroxine alone is adequate [82]. Treatment should be initiated in any infant with a positive screening result, right after confirmatory tests are drawn but before results are available [82,83].

Currently, only l-thyroxine tablets are approved for use in the United States. Thyroid suspensions prepared by individual pharmacies may result in unreliable dosing. In Europe, however, l-thyroxine drops have been successfully used [83]. The l-thyroxine tablet should be crushed, mixed with breast milk, formula or water and fed to the infant. The tablet should not be mixed with soy formula as this has been shown to interfere with absorption. One retrospective study of 78 patients with congenital hypothyroidism showed that infants fed soy formula took significantly longer to achieve a TSH under 10 mU/l (150 days versus 40 days) [84]. Should the infant require soy formula, l-thyroxine should be given halfway between feeds and thyroid function should be monitored carefully [80]. Some nutritional supplements or drugs are known to interfere with absorption of l-thyroxine.

These include:

• Soy protein formulas

• concentrated iron

• calcium, aluminum hydroxide

• Cholestyramine and other resins

• fiber supplements

• Sucralfate

Finally, prolonged heat exposure may reduce the efficacy of l-thyroxine tablets.

## Dosage

The dose and timing of thyroid hormone replacement are important in achieving optimal neurocognitive outcome. A delay in serum T4 normalization over one week can result in lower intelligence scores [81]. In one study, T4 normalization beyond two weeks resulted in patients scoring lower on behavioral and cognitive testing than patients who normalized in less than two weeks [85]. Thus, the goal of treatment should be to restore the serum T4 to > 129 mmol/L (> 10 μg/dl) as rapidly as possible. There is an inverse relationship between the starting l-thyroxine dose and the time to achieve the goal serum T4 concentration [85]. The recommended initial l-thyroxine dose set forth by the American Academy of Pediatrics (AAP) [82] and the European Society for Paediatric Endocrinology (ESPE) is 10-15 mcg/kg per day. In term infants this amounts to an average of 37.5 to 50 mcg per day [86]. In the aforementioned study, infants who received 50 mcg per day (12-17 mcg/kg/day) versus 37.5 mcg (10-15 mcg/kg/day) achieved higher performance scores for behavior, reading, spelling and math. Full scale IQ scores were 11 points higher in those started on 12- 17 mcg/kg/day [85]. Thus, for infants with low serum T4 levels, less than 5 μg/dl we would recommend using a dose of 12-17 mcg/kg/day, which is slightly above the higher end of the AAP recommended dose.

Infants with severe congenital hypothyroidism are at greater risk for developmental delay. This has been illustrated in studies done in both Europe, the United States and Canada [80,85][87-89]. Therefore, rapid replacement with adequate doses of l-thyroxine is particularly important. This point was illustrated in one study of 83 infants who were assigned to receive three different starting doses of thyroid hormone at birth. The first group received 6.0-8.0 mcg/kg/day, group 2 received a dose of 8.1-10.0 mcg/kg/day and Group 3, a dose of 10.1-15.0 mcg/kg/day. These infants were then followed for growth and intellectual outcome at four years of age. In this study, the infants with severe congenital hypothyroidism achieved the highest intellectual scores when started at the highest dose [90]. Another study done in 61 infants compared early versus late treatment with low versus high dosing. Results showed that in infants with severe congenital hypothyroidism, only those treated early (< 13 days) and with higher doses (>9.5 mcg/kg/day) achieved normal psychomotor development at 10-30 months of age [81].

It is important to note that the time for TSH normalization is inversely related to neurodevelopmental outcome [86]. One study done in 45 children compared intellectual outcome at 2 and 6 years of age with variance of serum T4 and TSH. These investigators found that scores on the mental development index (MDI) and verbal IQ were predicted by mean T4 and TSH during the first year of treatment [91]. Therefore it is important to closely monitor these infants and adjust the l-thyroxine dose frequently until the desired level is achieved.

## Treatment Goals

The treatment goals as outlined by the American Academy of Pediatrics (AAP) Update of newborn screening and therapy for congenital hypothyroidism [82] are similar to published European Society for Pediatric Endocrinology (ESPE) guidelines [86] and are as follows:

• Serum Free T4 or total T4 should be kept in the upper range of normal during the first year of life.

• Target values during the first year are 130 to 206 nmol/L (10-16 μg/dl) for the serum T4 and 18 to 30 pmol/L (1.4 to 2.3 ng/dl) for free T4.

• Serum TSH should be kept under 5 mU/L.

Infants who have serum T4 concentrations below 10 μg/dL in the first year of life accompanied by serum TSH concentrations above 15 mU/L have been shown to have lower IQ's than infants whose serum T4 concentrations are above 10 μg/dL [91]. Also, higher doses of l-thyroxine have been associated with higher intelligence quotients at 7 and 8 years of life, especially in the areas of verbal memory and verbal comprehension [92]. Thirdly, variations in serum T4 and TSH during the first year of life have been correlated with changes in mental development index and verbal intelligence quotient [91,93].

This would suggest that higher doses of l-thyroxine lead to better overall developmental outcomes. However, the last study also notes that children on high dose l-thyroxine treatment had significant problems with hyperactivity, delinquency and aggression [92]. Other studies have also shown that high serum T4 levels contribute to poorer attention in school aged children [94]. These highlight the dangers of overtreatment in congenital hypothyroidism.

One study followed children on high dose therapy till four years of age; this demonstrated that higher intelligence quotients were observed in patients started on 10-15 mcg/kg/day without adverse effects on growth and skeletal maturation [90]

## Recommended follow up

Clinical evaluation should be performed every few months during the first three years of life along with frequent measurements of serum T4 or free T4 and TSH. The American Academy of Pediatrics recommends the following monitoring schedule [86].

• At two and four weeks after the initiation of l-thyroxine treatment

• Every 1-2 months during the first 6 months of life

• Every 3-4 months between 6 months and three years of age

• Every 6-12 months thereafter until growth is complete

• Four weeks after any change in dose

More frequently if results are abnormal or non-compliance is suspected.

The serum T4 should normalize within one to two weeks and the serum TSH should become normal in most infants after one month of treatment. In some individuals, a high TSH (10-20 mU/L) may persist despite a normal serum T4 or vice versa [95]. In most cases this is due to under treatment, however there are some individuals who will have abnormal maturation of free T4 feedback control on TSH secretion [96]. This abnormality is thought to exist in about 10 percent of treated individuals with congenital hypothyroidism and may be due to resetting of the pituitary-thyroid feedback mechanism in utero [97]. In one study of 42 patients, the prevalence of pituitary thyroid hormone resistance was as high as 43 per cent in younger infants less than one year and decreased to 10 percent in children and adolescents[97]. This suggests that thyroid hormone resistance is more common in the younger age group and may resolve with age.

## Permanent versus transient hypothyroidism

Some patients with a positive newborn screen for congenital hypothyroidism have transient congenital hypothyroidism. Permanent congenital hypothyroidism can be assumed if:

• Ultrasonography or radionuclide imaging shows an absent or ectopic thyroid gland, consistent with athyreosis or thyroid dysgenesis.

• One can demonstrate dyshormonogenesis as discussed in the section on diagnosis.

• If at any time during the first year of life, the serum TSH rises above 20 mU/L due to undertreatment.

If permanent congenital hypothyroidism has not been established by two to three years of age the AAP and the ESPE recommend a 30 day trial off l-thyroxine therapy [86,98].

If a low serum T4 or free T4 and an elevated TSH are found, permanent congenital hypothyroidism is confirmed and the patient is restarted on therapy. If the serum T4 or freeT4 and TSH remain normal, the presumed diagnosis is transient congenital hypothyroidism and treatment is no longer needed. However, these patients must be followed closely and monitored for signs and symptoms of hypothyroidism such as constipation, slowing of growth or decreased mentation. If these appear then serum testing of thyroid function should be performed and if inconclusive, these patients should be continued to be followed closely with a low threshold for re-testing. Subjects with presumed transient hypothyroidism are vulnerable to recurrence during puberty and pregnancy and should be retested during these times. One novel approach is the use of recombinant TSH (rhTSH) to make the diagnosis of congenital hypothyroidism without requiring withdrawal of thyroid hormone. One study done on 10 children combined the use of ultrasound, scintigraphy after rhTSH, and percholorate discharge testing. This protocol resulted in the accurate diagnosis of permanent congenital hypothyroidism in 8 of 10 cases without stopping thyroxine. This shows that rhTSH may be of use in the future confirmation of permanent congenital hypothyroidism [99].

## Prognosis

Prior to the newborn screening era, when a diagnosis of congenital hypothyroidism was made after development of clinical manifestations, studies reported an inverse relationship between the age of diagnosis and IQ outcome. A study from Pittsburgh Children's Hospital showed that if thyroid hormone treatment was started between birth and 3 months of age, the mean IQ was 89 (range 64 to 107); if treatment was started between 3 and 6 months of age, the mean IQ was 71 (range 35 to 96), while if treatment did not start until after 6 months of age, the mean IQ dropped to 54 (range 25 to 80) [100]. A report from Sweden found that "in spite of an efficient National Health Care Program for infants, the diagnosis was delayed until after 3 months in 52 percent of cases"[1].

The advent of newborn screening programs in the mid-1970s allowed earlier detection and treatment of infants with congenital hypothyroidism. Such efforts have been successful in achieving a much-improved neurocognitive outcome. Despite this, however, not all studies report a completely normal outcome. In a recent review of 51 published reports of IQ outcome in infants with congenital hypothyroidism as compared to sibling or classmate control subjects, 18 found no significant IQ difference, while 33 found a significant difference, with IQ ranging between 5 and 25 points lower in infants with congenital hypothyroidism [80]. In evaluating important variables, there is evidence that age of onset of treatment, starting l-thyroxine treatment dose, and severity of hypothyroidism each plays an important role in neurocognitive outcome.

• **Age of onset of treatment **- A study from the French National Screening Program reported the effect of age of onset of thyroid hormone treatment, divided into four time periods, and IQ outcome [101]. In infants started on treatment after 30 days of age, mean global IQ = 109.8; if started between 22 and 30 days of age, mean IQ = 107.7, between 15 and 21 days, mean IQ = 115.3, and before 15 days of age, mean IQ = 119.2 (p=.008). Other programs, however, have not found an effect of age of onset of treatment. In a report from Australia, infants started after 14 days of age had a mean full scale IQ = 98.1, while those started before 14 days of age had a mean IQ = 99.6 (p = NS) [102]. It should be kept in mind that these were retrospective studies, and that comparisons of age of onset of treatment came about because early in the experience of screening programs infants generally were started on treatment at a later age, and then as screening programs became more experienced, the age of onset of treatment was lowered. A report from Italy compared infants started before and after 21 days of age, subdivided into two treatment groups: those started on 8.1-10 mcg/kg/day had a mean full scale IQ = 91 (> 21 days) vs. 96 (< 21 days). Those started on a higher dose, 10.1-15 mcg/kg/day) had a mean IQ = 98, identical in the group before or after 21 days of age [90]. Although these results did not reach statistical significance, at the lower starting dose there was a trend toward a better IQ with earlier treatment, whereas with the higher starting dose, the IQ in early vs. later treatment did not affect IQ outcome. Thus, there may have been factors other than age of onset of treatment that influenced IQ outcome, such as initial starting dose. It does appear that it is important to detect most cases and start treatment by 4 weeks of age. In our review of the literature, of 11 studies comparing starting treatment at an earlier age (12-30 days of life) vs. at a later age (> 30 days of life), infants started at the earlier age averaged 15.7 IQ points higher than infants started at a later age [80].< /21>

• **l-thyroxine starting dose **- At the time newborn screening programs were established in the mid-1970s, the recommended starting l-thyroxine dose was approximately 6-8 mcg/kg/day. With experience, it became evident that higher doses were needed to more rapidly correct the hypothyroxinemia and raise the serum T4 into the "target range" and lower serum TSH levels into the normal range (see Table 9). Early reports of psychometric outcome using a starting l-thyroxine dose of 6-8 mcg/kg/day showed a good outcome. The New England Congenital Hypothyroidism Collaborative reported a verbal IQ score of 109, performance IQ of 107, and full scale IQ of 109 at six years of age [103].

**Table 9:** Time course of normalization of serum T4 and TSH with initial l-thyroxine treatment dose (modified from: LaFranchi & Austin. *J Pediatr Endocrinol Metab *20:559-578, 2007[80].)

Screening Program	l-thyroxine dose (mcg/kg/day)	Time to serum T4 > 10 ug/dL (days)	Time to serum TSH < 9.1 mU/L (days)
Quebec	6	45-90	

Toronto	7-9	74	

France	8	15	60

New England	10	31	

US (Pennsylvania)	10-14	7	150

Italy	10-15	30	30

US (Oregon)	12-17	3	14

However, as the starting doses were increased, reaching the currently recommended 10-15 mcg/kg/day, programs were able to compare IQ outcomes at various starting l-thryoxine doses. A report from the Toronto screening program compared psychometric outcome in infants started on 6.4 mcg/kg/day vs. 9.0 mcg/kg/day [92]. Verbal IQ was 98.6 vs. 106.3 (p < .01), performance IQ was 103.8 vs. 108.2 (p = NS), and full scale IQ was 100.0 vs. 107.6 (p < .01) in the low vs. higher dose infants, respectively. A report from Italy compared psychometric outcome in infants started on three different l-thyroxine doses: a "low" dose 6-8 mcg/kg/day, an "intermediate" dose 8.1-10.0 mcg/kg/day, and a "high" dose 10.1-15 mcg/kg/day [90]. The verbal IQs in the three treatment groups were 92, 94, and 98, respectively (p = NS); performance IQs were 85, 95, and 98, respectively (p < .01), while the full scale IQs were 88, 94, and 98, respectively (p < .01). A report from the U.S. Northwest Regional Screening Program showed that infants started on a higher l-thyroxine dose (50 mcg/day, equivalent to 12-17 mcg/kg/day) had an IQ score 11 points higher than those started on the lower dose (37.5 mcg/day, equivalent to 9.4-12.4 mcg/kg/day) [85].

In our review of the literature, of ten studies examining the effect of different starting l-thryoxine doses on psychometric outcome, two reported no effect, six reported a 12.3 higher IQ with higher starting doses, while two actually reported a 8.6 point higher IQ with lower starting doses [80]. Overall, the preponderance of studies found that children started on the currently recommended l-thyroxine dose of 10-15 mcg/kg/day appear to achieve the best IQ outcome.

• **Severity of hypothyroidism** - Infants with congenital hypothyroidism have varying degrees of thyroid hormone deficiency; the severity of hypothyroidism likely is related to the underlying etiology (e.g, agenesis vs. hypoplasia/ectopia vs. dyshormonogenesis). It is important to bear in mind, however, that the degree of hypothyroidism is not simply related to the size of the residual thyroid gland. Some cases of dyshormonogenesis, with an enlarged gland, have severe hypothyroidism. Several screening programs have investigated psychometric outcome in relationship to severity of hypothyroidism, addressing the question of whether the most severely affected infants may have suffered prenatal damage that is not reversible even with early detection and treatment. The screening program in England, Wales, and Northern Ireland reported that infants with pre-treatment serum T4 < 3.3 ug/dL had a global IQ 11.6 points lower than infants with a serum T4 > 3.3 ug/dL [104]. The Quebec Screening Network compared IQ outcome in a cohort of infants with severe hypothyroidism, as assessed by a pre-treatment T4 < 2 ug/dL and an epiphyseal surface area < .05 cm2 vs. infants with more moderate hypothyroidism, with a pre-treatment T4 > 2 ud/dL and an epiphyseal surface area > .05 cm2 [105]. These infants were studied relatively early in the experience of their screening program, and so were started on a dose of 6 mcg/kg/day. The infants underwent serial psychometric testing; at age 12 years, the cohort with severe hypothyroidism had an IQ 16 points lower than the moderate group (p < .007). A report from the Northwest U.S. Regional Screening Program reported that infants with severe hypothyroidism (pre-treatment serum T4 < 2.2 ug/dL) had an IQ 11 points lower than a group with more moderate hypothyroidism (p < .05) [85].

Part of the explanation for a worse psychometric outcome in the most severely affected infants may be the lower starting l-thyroxine doses used in the early history of newborn screening. The Dutch newborn screening program investigated the effect of initial starting dose (< 9.5 or > 9.5 mcg/kg/day) and age of onset of treatment (< 13 days or > 13 days of age) in infants judged to have severe vs. milder hypothyroidism (pre-treatment serum free T4 0.21 ng/dL vs. 0.67 ng/dL, respectively) [106]. In the infants with severe hypothyroidism, psychometric testing at 10 to 30 months of age showed IQ 21 to 27 points lower in the groups treated with the lower starting dose and/or later age of onset; the group started on the higher dose and at the earlier age had the best IQ outcome (IQ = 125). On the other hand, all of the infants with milder hypothyroidism did well except the group treated with the lower dose and later age of onset, which had an IQ 22-25 points lower than the other groups [106]. These results support the concept of tailoring the initial starting l-thyroxine dose to the severity of hypothyroidism [107]. When the Quebec Screening Network used a higher starting dose, averaging 11.6 mcg/kg/day, they reported that psychometric testing did not show a difference in IQ in infants judged to have severe vs. more moderate hypothyroidism (107 vs. 110, respectively) [108].

• **Effect of lower serumT4 levels in the first two years of life and non-compliance** - Normal brain development depends on delivery of adequate thyroid hormone for the first two to three years of life. Low thyroid levels during this time may result in irreversible damage, whereas the effects of hypothyroidism after age 3 years generally are reversible when corrected. The New England Congenital Hypothyroidism Collaborative reported that a subgroup of 18 infants who had low serum T4 levels (average T4 8.6 ug/dL) and low l-thyroxine dosing (< 5 mcg/kg/day) with a history of poor compliance in the first three years of life, had a mean IQ of 87 [109]. The larger, adequately treated group, with a serum T4 in the target range (average T4 11.2 ug/dL), had an IQ score of 105. This study underscored the importance of frequent monitoring with dose adjustments to keep serum free T4 or T4 in the target range in the first two-three years of life.

The New England Congenital Hypothyroidism Collaborative also found that noncompliance beyond the first three years of life can affect cognitive performance. In a study of 14 year old adolescents with congenital hypothyroidism, the investigators made home visits without forewarning. Testing showed that 44% were inadequately treated, as judged by serum TSH > 15 mU/L, with serum T4 < 6.6 ug/dL (< 85 nmol/L) in the majority [110]. Psychometric testing showed a mean IQ of 106. Compliance with thyroid hormone treatment was stressed (the dose was not changed), and one to two years later, when thyroid testing was now improved, repeat psychometric testing showed a mean IQ of 112 (p < .002). This study showed that noncompliance in adolescents is common, and that improvement in compliance and thyroid levels was associated with improved cognitive function.

## Unresolved questions

The underlying etiology of thyroid dysgenesis remains largely unknown. As described in the **Etiology, Epidemiology **and **Genetic Counseling **sections, while dysgenesis appears sporadic, there are clues to some familial/genetic factors. Approximately 2 percent of cases of thyroid dysgenesis have been shown to result from mutations in genes that code for transcription factors important in thyroid gland development, such as *TTF-2*, *PAX-8*, and *NKX2*. The higher incidence in certain racial and ethnic groups, in preterm infants, in twin and multiple births, and in older mothers points toward genetic or perhaps epigenetic factors that have yet to be discovered. In addition, the approximate 2:1 female:male ratio overall, more apparent with ectopic glands than with thyroid agenesis[111] is unexplained. Again, this finding points to undiscovered genetic factors, perhaps linked to autoimmuity, which is usually more common in females.

The explanation for the apparent increase in incidence of congenital hypothyroidism over the last 20 years is unclear. Is the increase real, or is it the result of changes in screening program test cutoffs, such that infants with milder cases of hypothyroidism are now being detected? Or, are there other factors, such as changes in the racial/ethnic population mix that contribute to the increase? It is also unclear whether the additional infants now being detected, including those with mild hypothyroidism and those with "delayed TSH rise" will have permanent or transient hypothyroidism.

Management of antenatal hypothyroidism as it relates to psychometric outcome is an open question. As discussed under **Antenatal diagnosis**, it is relatively rare to discover hypothyroidism *in utero*. When such cases are discovered, clinicians may feel pressure to treat the hypothyroidism with amniotic fluid injections of l-thyroxine. However, given the good neurocognitive outcome in infants with congenital hypothyroidism detected by newborn screening programs and started on thyroid hormone treatment in the first 2 to 4 weeks of life (who are not treated *in utero*), and the potential risks of intra-amniotic fluid injections and fetal cord blood sampling, treatment after birth may be a reasonable course of management. It is unlikely that there will ever be enough cases to perform randomized clinical trials to address this question. Psychometric testing in infants both treated and not treated antenatally, however, may provide useful information. A case can be made to treat hypothyroidism antenatally if a significant goiter is present.

## Competing interests

The authors declare that they have no competing interests.

## Consent

Written informed consent was obtained from the parent of the patient for publication and accompanying images. A copy of the written consent is available for review by the Editor-in-Chief of this journal.

## Authors' contributions

MR has researched and written the sections on clinical features, etiology and management and built tables 2, 3, 4, and 6. The remaining sections and tables were written by SL. Both authors read and approved the manuscript.
